# A phase III, open label, randomized multicenter controlled trial of oral versus intravenous treosulfan in heavily pretreated recurrent ovarian cancer: a study of the North-Eastern German Society of Gynecological Oncology (NOGGO)

**DOI:** 10.1007/s00432-016-2307-0

**Published:** 2016-11-28

**Authors:** Jalid Sehouli, Oliver Tomè, Desislava Dimitrova, Oumar Camara, Ingo Bernhard Runnebaum, Hans Werner Tessen, Beate Rautenberg, Radoslav Chekerov, Mustafa Zelal Muallem, Michael Patrick Lux, Tanja Trarbach, Gerald Gitsch

**Affiliations:** 1 Gynecology and Gynecologic Oncology, Charité University Medicine Campus Virchow, Augustenburger Platz 1, 13353 Berlin, Germany; 2 Gynecologic Cancer Centre Karlsruhe, St. Vincentius Clinics, Karlsruhe, Germany; 3 Centre for Gynecology, Hufeland Clinic, Bad Langensalza, Germany; 4 Clinic for Gynecology and Obstetrics, University Clinic, Jena, Germany; 5 Oncology Practise, Goslar, Germany; 6 Gynecology and Gynecologic Oncology, University Clinic, Freiburg, Germany; 7 Department of OOGYN, Gyncecological University Cancer Center of Franconia, University Hospital Erlangen, CCC Erlangen-EMN, Erlangen, Germany; 8 iOMEDICO Clinical Research, Freiburg, Germany

**Keywords:** Treosulfan, Oral versus intravenous, Ovarian cancer, Recurrent

## Abstract

**Objective:**

In recurrent ovarian cancer (ROC), there is a high demand on effective therapies with a mild toxicity profile. Treosulfan is an alkylating agent approved as oral (p.o.) and intravenous (i.v.) formulation for the treatment of recurrent ovarian cancer. Data on safety and efficacy for either formulation are rare. For the first time we conducted a randomized phase III study comparing both formulations in women with ROC.

**Methods:**

Patients having received at least two previous lines of chemotherapy were randomly assigned to one of two treatment arms: treosulfan i.v. 7000 mg/m^2^ d1 q4w or treosulfan p.o. 600 mg/m^2^ d1-28 q8w. Primary endpoint was safety regarding hematological and gastrointestinal toxicity grade III/IV, secondary endpoints were other toxicities, clinical benefit rate (CBR), time to progression (TTP), overall survival (OS) and quality of life.

**Results:**

250 patients were treated with treosulfan i.v. (128) or treosulfan p.o. (122). In general treosulfan therapy was well tolerated in both treatment arms. Leukopenia grade III/IV occurred significantly more frequently in the p.o. arm (3.9% i.v. arm, 14.8% p.o. arm, *p* = 0.002). Other toxicities were similar in both arms. CBR was comparable between arms (41.4% i.v. arm, 36.9% p.o. arm). No difference in TTP (3.7 months i.v. arm, 3.5 months p.o. arm) or OS (13.6 months i.v. arm, 10.4 months p.o. arm, *p* = 0.087) occurred.

**Conclusions:**

Given the safety and efficacy results treosulfan is an acceptable option for heavily pretreated OC patients. Regarding the toxicity profile the i.v. application was better tolerated with less grade III and IV toxicities.

**Electronic supplementary material:**

The online version of this article (doi:10.1007/s00432-016-2307-0) contains supplementary material, which is available to authorized users.

## Introduction

In Europe, ovarian cancer is the fifth most common cancer and one of the five leading malignancies responsible for cancer-related deaths among females (Ferlay et al. 2013). As the results of EUROCARE-5 study demonstrated the European mean age-standardized 5-year survival for ovarian cancer is low-37.6% (De Angelis et al. 2014). About 66,000 women in Europe were diagnosed with ovarian cancer and estimated 42,000 died of this disease in 2012 (Ferlay et al. 2013). Primary treatment of ovarian cancer includes cytoreductive surgery followed by systemic chemotherapy. Standard first-line chemotherapy is based on platinum and taxanes—eventually in combination with bevacizumab—which achieves high response rates up to 80% (McGuire et al. 1996; Pujade-Lauraine et al. 2010). However, in the majority of patients the disease recurs and further therapy is required. Drug-resistance to platinum-based chemotherapy is one of the most difficult clinical situations because the effect of the current therapies is very limited and the outcome is poor. There is a need for implementation of new chemotherapeutic agents for further treatment. Treosulfan (Ovastat^®^) is a bifunctional prodrug of an alkylating cytotoxic agent that is licensed in several European countries for the treatment of ROC (Gropp et al. 1998; Hilger et al. 2000). Treosulfan also shows activity in other solid tumors and hematologic malignancies (Boztug et al. 2014; Köpf-Maier and Sass 1996). It is known for its modest toxicity profile as demonstrated in previous phase II and III studies (Reed et al. 2006; Kledsen et al. 1998). With a relative bioavailability of 97%, treosulfan p.o. has shown a nearly equivalent bioavailability (AUC_oral_ = 82.1 ± 39.4 µg/ml h) compared to treosulfan i.v. (AUC_i.v._ = 85.4 ± 30.3 µg/ml h) in ovarian cancer patients (Hilger et al. 2000). Till now data on safety and efficacy for either formulation in heavily pretreated patients with ovarian cancer are rare. Therefore, for the very first time, we conducted a randomized phase IIIb study comparing the intravenous versus oral formulation of treosulfan in women with recurrent ovarian cancer.

## Patients and methods

This open label, randomized, controlled, multicenter phase IIIb trial was conducted at 30 institutions in Germany. Patients were eligible for enrollment, if they had histologically confirmed ROC and had received at least two previous lines of chemotherapy. After inclusion of 18 patients an amendment inured, stating that induction and re-induction of platinum-containing pretreatments were counted as one treatment line. A further amendment was implemented after the inclusion of 85 patients stating that also patients with more than two previous treatment lines could be included, and induction and re-induction therapy were counted as separate treatment lines. For data analysis, induction and re-induction therapy lines were counted as one therapy line. Written informed consent was given by all patients before enrollment. For patient inclusion following criteria needed to be met: (1) two-dimensional measurable tumor lesion or progressive disease evaluable by increased CA125 (>100 U/ml); (2) life expectancy of at least 3 months; (3) Karnofsky index >50%; (4) adequate organ functions, defined as leukocyte count ≥3.5 cells × 10^9^/l, platelet count ≥100 × 10^9^/l, creatinine and total bilirubin 1.25 × the upper limit of the normal range or less; (5) no prior treatment with treosulfan; (6) no second malignancy (except basilioma or cervical cancer in situ); (7) no ascites/pleural effusion without evaluable or measurable tumor lesion or not elevated CA125; (8) no concurrent radiotherapy or antineoplastic therapy.

Patients were randomly assigned to one of the two treatment arms: treosulfan i.v. 7000 mg/m^2^ d1 q4w or treosulfan p.o. 600 mg/m^2^ d1-28 q8w. Treatment was continued until tumor progression, unacceptable toxicity or treatment delay for more than 2 weeks. Patients with a complete remission received optional chemotherapy for two more months before therapy was terminated. Supportive care was allowed and given individually according to clinical requirements. Patients in both arms needed to have a leukocyte count of at least 3500 cells/µl and a platelet count of at least 100,000/µl to receive subsequent cycles of chemotherapy. Treosulfan dose had to be reduced for 1000 mg/m^2^ body surface for i. v. administration or one capsule for oral administration in the next treatment cycle, if leukocytes dropped below 1000 cells/µl or platelets dropped below 25,000/µl upon administration of treosulfan. Re-escalation was not allowed. For patients with renal insufficiency receiving treosulfan i.v., treatment-dose was reduced to 6000 mg/m^2^, if creatinine clearance was 20–40 ml/min and to 5000 mg/m^2^ if creatinine clearance was less than 20 ml/min. The primary endpoint of the study was the comparison of safety of both schemes regarding hematological and gastrointestinal toxicity grade III and IV. Adverse events were assessed continuously by patient questioning, physical examination and evaluation of laboratory results, and graded according to the National Cancer Institute Common Toxicity criteria (CTC) version 2.0. Secondary endpoints were comparison of other adverse events, clinical benefit rate (CBR), time to progression (TTP), overall survival (OS) and quality of life. Response was evaluated by assessment of two-dimensional measurable lesions and determination of CA125 levels. Lesions were examined every 3 months using imaging procedures. CA125 levels were determined every month. A complete response was defined as complete disappearance of tumor lesions. A partial response was defined as 50% or more decrease in tumor size. Assessments had to be confirmed after at least 4 weeks. No change/stable disease was defined as less than 50% decrease or less than 25% increase in tumor size or of reference metastasis. Progressive disease was defined as appearance of any new lesions or growth of more than 25% or increase in CA125 level by more than 25%. This increase had to be confirmed after at least 2 weeks. TTP was defined as the time from the date of randomization to the date of first documented progression. Patients not experiencing PD were censored with the last date of either tumor evaluation, measurement of CA125 or with the start of a new therapy. Survival was defined as the time from date of therapy start to date of death. Patients who were alive at the end of the study were censored at the last date where they were known to be alive. Platinum-sensitivity was defined by a relapse-free period of more than 6 months following platinum-based chemotherapy. Platinum-sensitivity was calculated using the relapse-free interval from end of the last platinum-containing therapy until start of the following therapy (Hanker et al. 2012). For the analyses of treatment duration, every cycle started was calculated as a full cycle. Quality of life was assessed using the EORTC QLQ-C30 questionnaire. Patients were asked to complete the questionnaire before first cycle of study therapy and consecutively during treatment every other month. The last questionnaire was completed at the end of treatment visit. Once patients discontinued therapy because of complete remission or intolerable toxicity, disease status was assessed every 3 months. After disease progression follow-up was only performed on survival data.

## Statistical analysis

The sample size was planned to detect a 15% difference in the incidence of grade 3/4 adverse events of hematological or gastrointestinal origin between the two treatment arms. An event was assumed to occur with a probability of 25%. Calculation was based on a two-sided test with *α* = 0.05 and 80% power. As no clear hypothesis was stated, all *p* values are explorative. There was no adjustment for multiple testing. A total number of 270 patients (129 per treatment arm plus 4% dropouts) were planned to be recruited. All analyses were based on the safety population including all patients who received at least one dose of study medication. Differences in proportions of adverse events and response were analyzed using a two-sided Fisher’s exact test or two-sided *χ* ^2^ test. Clinical benefit rate (CBR) was defined as sum of complete remission (CR), partial remission (PR) and no change (NC) as best response (CBR = CR + PR + NC). Time to progression (TTP) and overall survival time (OS) were estimated by use of Kaplan–Meier, and differences were compared using the log-rank test. Subgroup analyses were performed retrospectively. Hazard ratios were calculated using cox and logistic models. Effects of possible confounders were estimated using multivariate cox or logistic regression modeling. For the calculation of quality of life scores, scoring manuals were applied. All analyses were performed using STATISTICA version 10 or R 2.15.1.

## Results

### Patient characteristics

**Fig. 1:**
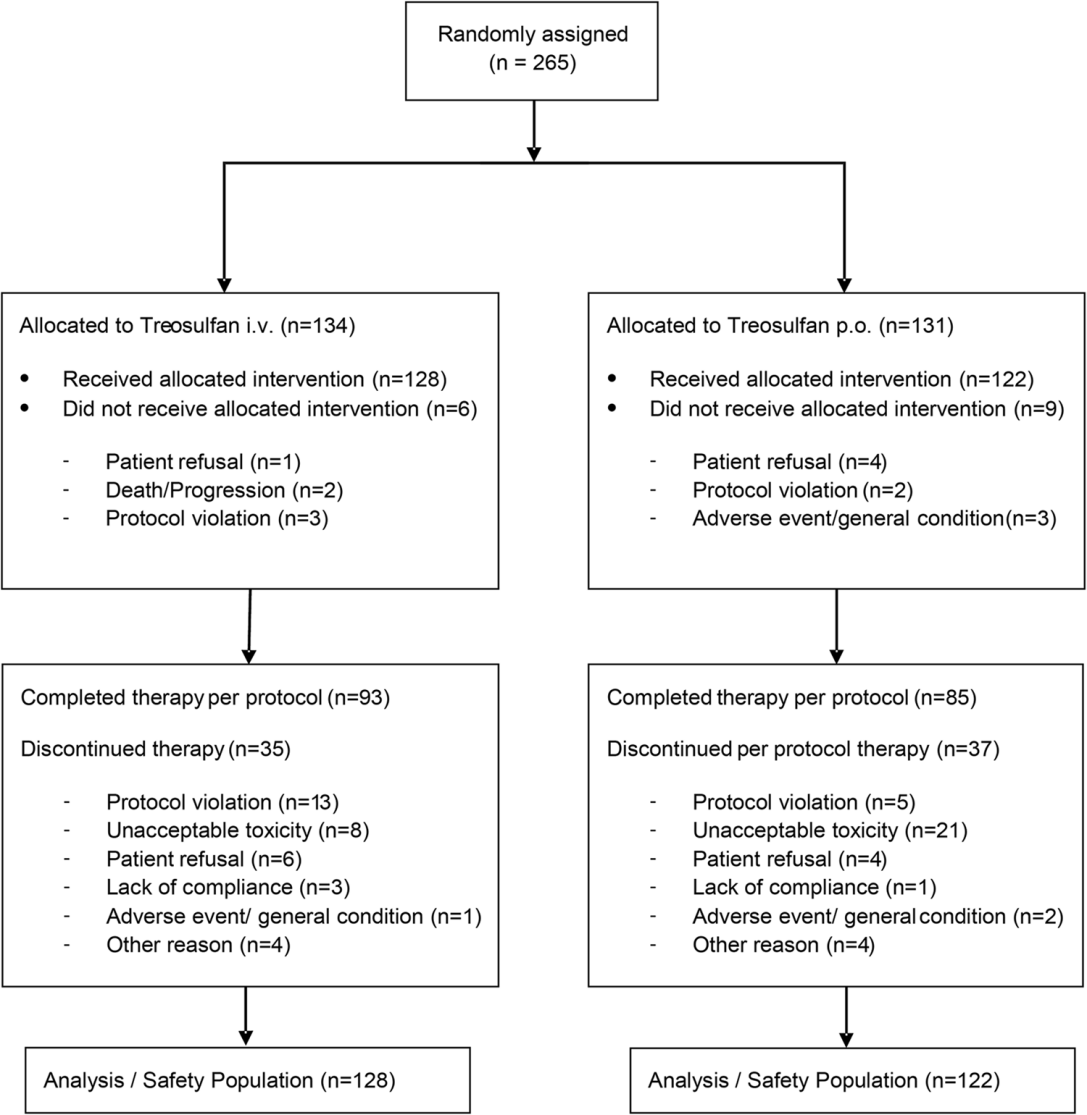
CONSORT diagram. i.v., intravenous; p.o., per os/oral

Between November 2002 and January 2014, 265 patients were enrolled at 30 study sites. Patient consort diagram is shown in Fig. 1. 250 patients (128 treosulfan i.v. and 122 treosulfan p.o.) received at least one dose of treosulfan and were evaluable (safety set). Patient characteristics are shown in Table 1. There were no substantial differences between both treatment arms. Patients received two previous chemotherapies in median. As first-line therapy 82% of patients received a platinum-containing regimen combined with paclitaxel. As second-line therapy topotecan or a platinum-containing regimen were administered in about 45% of patients. The median number of concomitant diseases was three. Most important concomitant diseases are listed in Table 1.

**Table 1:** Baseline characteristics

	Treosulfan i.v. *n* = 128	Treosulfan p.o. *n* = 122
Age in years at therapy start [median (range)]	63 (32–84)	64 (29–87)
Karnofsky index		
100%	37 (28.9)	36 (29.5)
90%	33 (25.8)	27 (22.1)
80%	33 (25.8)	36 (29.5)
≤70%	12 (9.4)	11 (9.0)
Not specified/missing	13 (10.2)	12 (9.8)
FIGO stage at initial diagnosis		
I	5 (3.9)	5 (4.1)
II	5 (3.9)	2 (1.6)
III	73 (57.0)	60 (49.2)
IV	30 (23.4)	29 (23.8)
Not specified	15 (11.7)	26 (21.3)
Relapse-free interval after last platinum-containing therapy		
<6 months	68 (53.1)	65 (53.3)
>6 months	60 (46.9)	57 (46.7)
Previous therapies		
Surgery (initial)	127 (99.2)	122 (100.0)
Radiotherapy	9 (7.0)	7 (5.7)
Number of previous chemotherapy lines [median (range)]	2 (1–7)	2 (1–6)
2 previous lines	81 (63.3)	80 (65.6)
≥2 previous lines	47 (36.7)	42 (34.4)
Concomitant diseases	3 (1–13)	3 (1–11)
Gastrointestinal disorders	62 (48.4)	68 (55.3)
Vascular disorders	63 (49.2)	62 (50.4)
Hypertension	42 (32.8)	49 (39.8)
Thromboembolism	12 (9.4)	7 (5.7)
Metabolism/nutrition and endocrine disorders	59 (46.1)	62 (50.4)
Diabetes	8 (6.3)	11 (8.9)
Respiratory disorders	28 (21.9)	23 (18.7)
Thromboembolism	5 (3.9)	6 (4.9)
Cardiac disorders	12 (9.4)	20 (16.3)
Cardiac failure	4 (3.1)	4 (3.3)
Coronary artery disease	2 (1.6)	3 (2.4)

Data are presented as *n* (%) unless specified separately; FIGO, International Federation of Gynecology and Obstetrics; study sites could choose to document either FIGO or TNM stage, TNM documentation was translated into FIGO (according to NCCN guidelines version 3.2014 on epithelial ovarian cancer)

### Treatment

The mean number of administered treatment cycles was 4.4 (95% CI 3.7–5.1; median 3.0) in the i.v. arm and 2.3 (95% CI 1.9–2.8; median 2.0) in the p.o. arm. Considering that i.v. and p.o. treatment cycles are of different length (28 days for the i.v. arm and 56 days for the p.o. arm) treatment duration was comparable in both treatment arms. 64% (160 of 250) of patients continued study treatment until disease progression as scheduled in the protocol. Unforeseen treatment discontinuations were limited and comparable in both treatment arms (27.3% in the i.v. arm and 30.3% in the p.o. arm). The proportion of discontinuations due to toxicity was significantly higher in the p.o. treatment arm (17.2%) than in the i.v. treatment arm (6.3%; *p* = 0.0094) (Fig. 1). The median dose intensity in the i.v. arm was 97.8% (25–75% quartile, 90.0–100.8%) and 99.6% on the p.o. arm (25–75% quartile, 84.0–124.4%).

### Adverse events/toxicity

Adverse events occurred in 91.2% of patients (90.6% in the i.v. arm and 91.8% in the p.o. arm). Toxicities of grade III/IV were rare. 25.0 and 30.3% of patients in the i.v. and p.o. arm experienced serious adverse events, respectively. Frequently reported hematological and non-hematological toxicities of all grades are listed in Table 2. Hematological and gastrointestinal toxicities were the main grade III/IV toxicities and were observed in 17.6% (12.5% in the i.v. arm and 22.1% in the p.o. arm) and 18.0% (17.9% in the i.v. arm and 18.0% in the p.o. arm) of patients, respectively. The severity of hematological events differed between treatment arms. Although the total number of reported hematological events of all grades was similar in both arms (40.6% in the i.v treatment arm, 39.3% in the p.o. treatment arm), grade III/IV events occurred more often in the p.o. arm (22.1% vs.12.5% in the i.v. arm). The frequency of grade III/IV leukopenia was significantly higher in the p.o. treatment arm than in the i.v. arm (14.8% in the p.o. arm and 3.9% in the i.v. arm, *p* = 0.0038). However, only one patient in the p.o. arm experienced a febrile neutropenia grade III. In addition to hematological and gastrointestinal toxicities, constitutional symptoms and pain were recorded most frequently. Alopecia occurred in 19 (7.6%) of patients. Almost all cases were grade I events, only one patient suffered from alopecia grade II. No grade III alopecia occurred. Except the above mentioned, no differences in hematological and non-hematological toxicities of any grade between both arms were observed. 17 patients (11 in the i.v. arm and 6 in the p.o. arm) died during study period. In all cases, death was concerned as not related to study medication. Underlying malignant disease was stated as cause of death for ten patients in the i.v. arm and five patients in the p.o. arm. Two patients, one in the i.v arm and one in the p.o. arm died of thromboembolic events (pulmonary embolism in the p.o. arm and stroke in the i.v. arm) not considered to be caused by the study medication.

**Table 2:** Hematological and non-hematological toxicities all grades, highest grade per patient

	Treosulfan i.v. (*n* = 128)					Treosulfan p.o. (*n* = 122)				
	Grade 1	Grade 2	Grade 3	Grade 4	Total	Grade 1	Grade 2	Grade 3	Grade 4	Total
Patients with any adverse event					116 (90.6)					112 (91.8)
Patients with SAE					32 (25.0)					37 (30.3)
Hematological toxicities										
Blood/bone marrow	10 (7.8)	26 (20.3)	15 (11.7)	1 (0.8)	52 (40.6)	6 (4.9)	15 (12.3)	21 (17.2)	6 (4.9)	48 (39.3)
Leukocytes (total WBC)*	2 (1.6)	16 (12.5)	4 (3.1)	1 (0.8)	23 (18.0)	2 (1.6)	13 (10.7)	18 (14.8)	–	33 (27.0)
Platelets	11 (8.6)	5 (3.9)	8 (6.2)	–	24 (18.8)	8 (6.6)	5 (4.1)	8 (6.6)	2 (1.6)	23 (18.9)
Hemoglobin	6 (4.7)	17 (13.3)	4 (3.1)	–	27 (21.1)	1 (0.8)	9 (7.4)	3 (2.5)	2 (1.6)	15 (12.3)
Gastrointestinal toxicities										
Gastrointestinal	30 (23.4)	26 (20.3)	14 (10.9)	9 (7.0)	81 (63.3)	31 (25.4)	23 (18.9)	17 (13.9)	5 (4.1)	76 (62.3)
Nausea	27 (21.1)	16 (12.5)	2 (1.6)	–	46 (35.9)	30 (24.6)	5 (4.1)	4 (3.3)	–	39 (32.0)
Vomiting	7 (5.5)	15 (11.7)	3 (2.3)	–	25 (19.5)	8 (6.6)	16 (13.1)	2 (1.6)	1 (0.8)	27 (22.1)
Constipation	14 (10.9)	8 (6.2)	1 (0.8)	1 (0.8)	25 (19.5)	11 (9.0)	6 (4.9)	2 (1.6)	–	19 (15.6)
Diarrhea	11 (8.6)	4 (3.1)	–	–	16 (12.5)	15 (12.3	4 (3.3)	–	–	19 (15.6)
Ileus	–	–	9 (7.0)	9 (7.0)	18 (14.0)	–	–	6 (4.9)	4 (3.3)	10 (8.2)
Stomatitis/pharyngitis	3 (2.3)	4 (3.1)	–	–	7 (5.5)	5 (4.1)	–	–	–	5 (4.1)
Other toxicities										
Fatigue	24 (18.8)	13 (10.2)	1 (0.8)	–	38 (29.7)	24 (19.7)	9 (7.4)	3 (2.5)	–	36 (29.5)
Pain	24 (18.8)	23 (18.0)	3 (2.3)	–	50 (39.1)	24 (19.7)	17 (13.9)	4 (3.3)	–	45 (36.9)
Abdominal pain or cramping	12 (9.4)	7 (5.5)	2 (1.6)	–	21 (16.4)	18 (14.8)	7 (5.7)	2 (1.6)	–	27 (22.1)
Arthralgia	4 (3.1)	3 (2.3)	–	–	7 (5.5)	4 (3.3)	3 (2.5)	–	–	7 (5.7)
Headache	5 (3.9)	1 (0.8)	–	–	6 (4.7)	5 (4.1)	2 (1.6)	–	–	7 (5.7)
Myalgia	6 (4.7)	2 (1.6)	–	–	8 (6.2)	2 (1.6)	–	–	–	2 (1.6)
Dermatology/skin	21 (16.4)	4 (3.1)	1 (0.8)	–	26 (20.3)	18 (14.8)	5 (4.1)	1 (0.8)	–	24 (19.7)
Alopecia	8 (6.2)	1 (0.8)	–	–	9 (7.0)	10 (8.2)	–	–	–	10 (8.2)
Pulmonary	5 (3.9)	15 (11.7)	4 (3.1)	–	24 (18.8)	4 (3.3)	17 (13.9)	4 (3.3)	1 (0.8)	26 (21.3)
Dyspnea	–	12 (9.4)	3 (2.3)	–	15 (11.7)	–	16 (13.1)	1 (0.8)	–	17 (13.9)
Neurology	21 (16.4)	4 (3.1)	4 (3.1)	1 (0.8)	30 (23.4)	14 (11.5)	5 (4.1)	–	–	19 (15.6)
Neuropathy sensory	9 (7.0)	–	–	–	9 (7.0)	8 (6.6)	3 (2.5)	–	–	11 (9.0)
Infection/febrile neutropenia	6 (4.7)	12 (9.4)	3 (2.3)	1 (0.8)	22 (17.2)	4 (3.3)	6 (4.9)	6 (4.9)	–	16 (13.1)
Infection without neutropenia	4 (3.1)	9 (7.0)	–	–	13 (10.2)	3 (2.5)	6 (4.9)	2 (1.6)	–	11 (9.0)
Renal/genitourinary	4 (3.1)	7 (5.5)	1 (0.8)	–	12 (9.4)	6 (4.9)	4 (3.3)	4 (3.3)	1 (0.8)	15 (12.3)
Cardiovascular (general)	6 (4.7)	2 (1.6)	2 (1.6)	1 (0.8)	11 (8.6)	3 (2.5)	5 (4.1)	2 (1.6)	2 (1.6)	12 (9.8)
Musculoskeletal	3 (2.3)	5 (3.9)	–	–	8 (6.2)	4 (3.3)	2 (1.6)	2 (1.6)	1 (0.8)	9 (7.4)

Data are presented as *n* (%), adverse events were graded according to the National Cancer Institute Common Toxicity Criteria version 2.0

* Events occurring with a frequency >5% in any grade are listed

### Efficacy

**Fig. 2:**
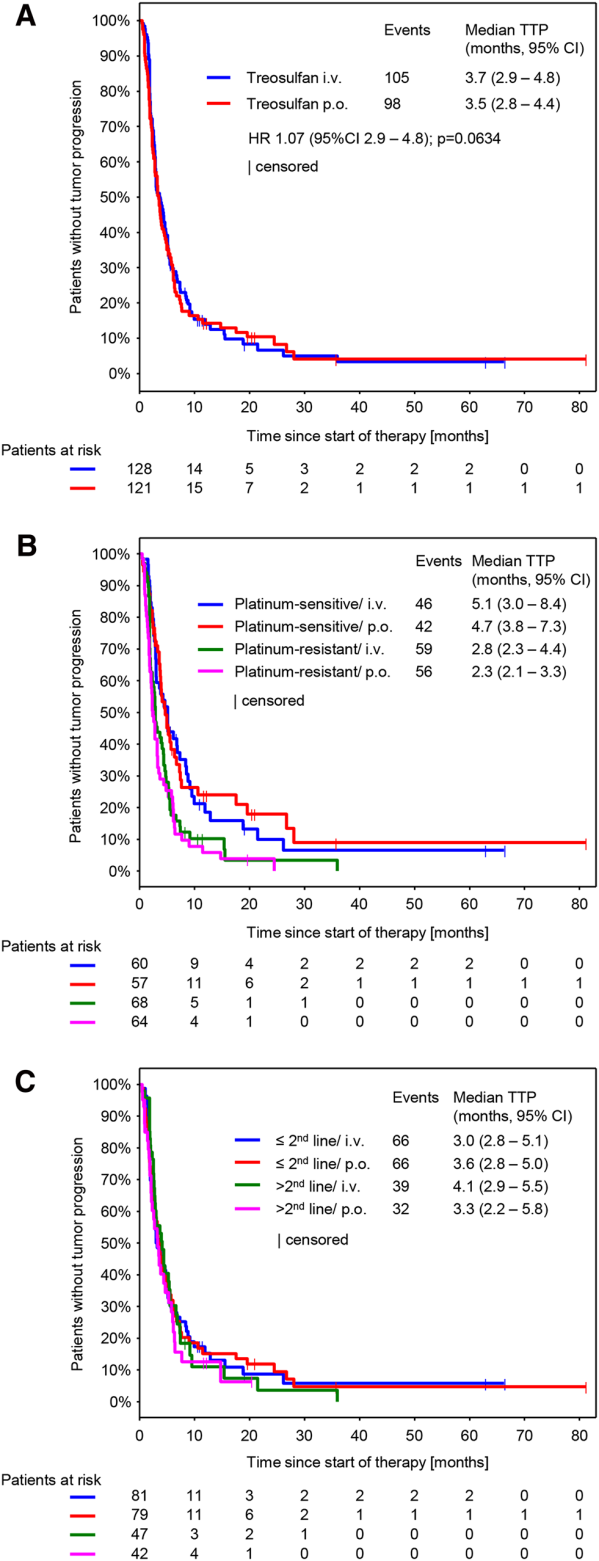
Time to progression (TTP) (**a**) By treatment arm (**b**). By platinum sensitivity and treatment arm (**c**) By number of previous therapy lines and treatment arm. *HR* Hazard ratio

**Fig. 3:**
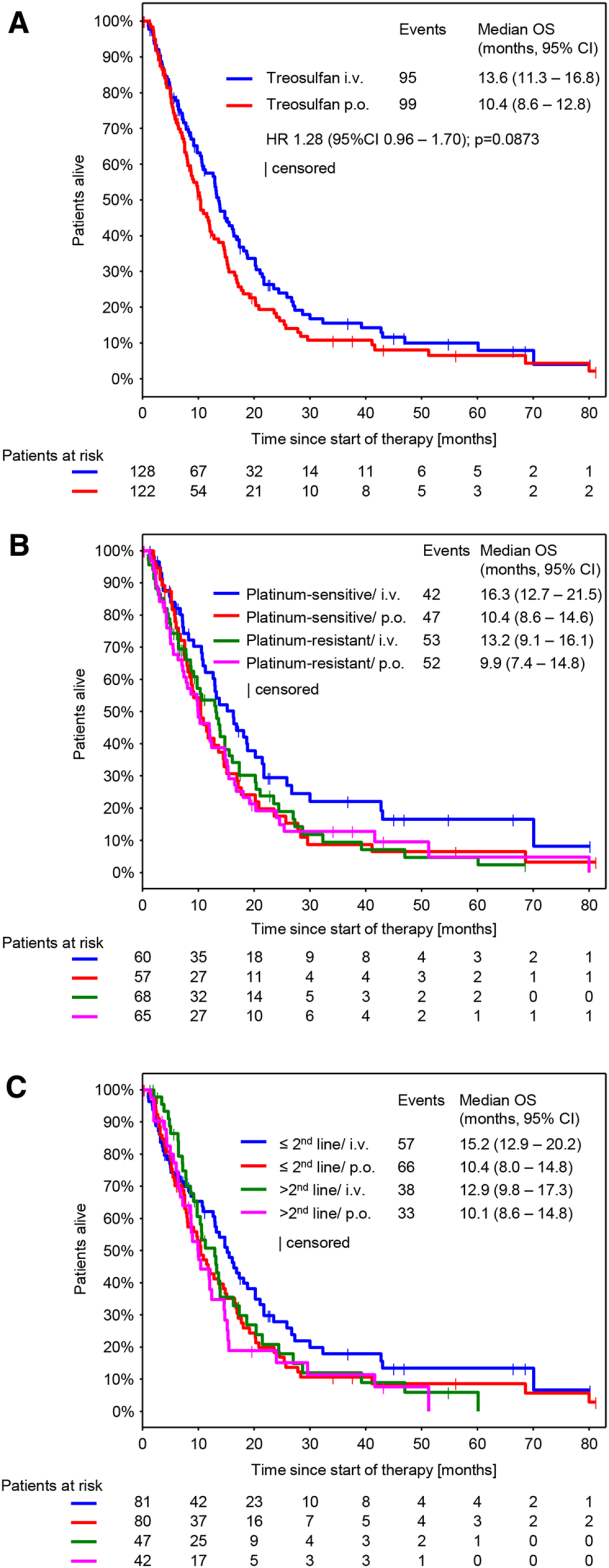
Overall survival (OS) (**a**) By treatment arm (**b**). By platinum sensitivity and treatment arm (**c**) By number of previous therapy lines and treatment arm. *HR* Hazard ratio; *TTP* Time to progression

Response was evaluable in 212 patients (111 in the i.v. arm and 101 in the p.o. arm). Non-evaluable patients mainly resulted from early treatment discontinuations, when no response assessment was performed. CBR was 39.2% (95% CI 33.2–45.6) and was comparable between both arms (treosulfan i.v.: 41.4, 95% CI 32.9–50.5; treosulfan p.o.: 36.9, 95% CI 28.5–46.1; *p* = 0.6063) (Table 3). The median follow-up was 8.6 months (10.0 months in the i.v. arm and 7.6 months in the p.o. arm). We could not detect any significant difference in the median TTP and median OS between both treatment arms (Figs. 2a, 3a). However, a nonsignificant trend toward longer survival in the i.v. arm was observed. Median TTP was 3.7 months (95% CI 2.9–4.8) in the i.v. treatment arm and 3.5 months (95% CI 2.8–4.4) in the p.o. treatment arm (Fig. 2a; HR 1.07, 95% CI 0.81–1.41, *p* = 0.634). Median OS was 13.6 months (95% CI 11.3–16.8) in the i.v. treatment arm and 10.4 months (95% CI 8.6–12.8) in the p.o. treatment arm (Fig. 2b; HR 1.28, 95% CI 0.96.1.70, *p* = 0.0873).

**Table 3:** Response evaluation

CBR (95% CI)	Treosulfan i.v. *n* = 128	Treosulfan p.o. *n* = 122	Total
	53 (41.4 (32.9 – 50.5))	45 (36.9 (28.5 – 46.1))	98 (39.2 (33.2 – 45.6))
CR	5 (3.9)	1 (0.8)	6 (2.4)
PR	16 (12.5)	15 (12.3)	31 (12.4)
NC	32 (25.0)	29 (23.8)	61 (24.4)
PD	58 (45.3)	56 (45.9)	114 (45.6)
NE	17 (13.3)	21 (17.2)	38 (15.2)

Best response per patient. Data are presented as *n* (%)

*CBR* Clinical benefit rate; *CR* Complete response; *PR* Partial response; *NC* No change; *PD* Progressive disease; *NE* Not evaluable

Subgroup analyses on efficacy parameters were performed using three models, each of them exploring the impact of the following covariates on CBR, TTP and OS: route of administration (p.o. vs. i.v.), platinum-sensitivity (platinum-sensitive vs. platinum-resistant), number of previous therapy lines (>2 lines vs. ≤2 lines) and Karnofsky index (<90 vs. ≥90) (S1 A-C). The model revealed significant differences for the following subgroups: Platinum-sensitive patients showed a higher CBR (47.9% vs. 31.6; *p* = 0.003) (S1 A) and developed a longer TTP (5.0 vs. 2.8 months; *p* < 0.0001) (Fig. 2b and S1 B) compared to platinum-resistant patients. In the subgroup of Karnofsky index, the model revealed a significant longer TTP and OS for patients with a Karnofsky index ≥90 (TTP: 4.3 vs. 3.0, *p* = 0.039 and OS: 15.1 vs. 8.6, *p* < 0.0001) (S1 B, C). For platinum-sensitivity, no differences in OS were observed. The model did not show significant differences for any efficacy parameter for the subgroup of number of previous therapy lines and the subgroup of route of administration.

58% of patients received at least one further therapy after completion of study therapy. 11.6% of subsequent therapies were platinum based.

### Quality of life/patient reported outcome

Questionnaire compliance was comparable in both treatment arms. Baseline questionnaires were available for 81.3% of patients in the i.v. treatment arm and from 82.0% of patients in the p.o. treatment arm. Significant differences in global health, fatigue as well as in gastrointestinal and hematological subscales were detected neither between the treatment arms nor over time during the first 6 months of treatment (S2 A-F).

## Discussion

As most ovarian cancer patients suffer from relapse, treatment of recurrent ovarian cancer remains a major issue in the clinical management of ovarian cancer. Standard chemotherapies are either platinum-based regimens in combination with agents such as liposomal doxorubicin, gemcitabin, paclitaxel and bevacizumab or olaparib (Pujade-Lauraine et al. 2010; Aghajanian et al. 2012; Oza et al. 2015) or non-platinum-based regimens such as liposomal doxorubicin, paclitaxel, topotecan and gemcitabine combined with targeted therapies like bevacizumab (Meier et al. 2009; Monk et al. 2010; Pujade-Lauraine et al. 2014; Sehouli et al. 2008). In general, patients with more than two previous lines of chemotherapy have been excluded from these trials, so translation of the results on heavily pretreated patients is limited. Various randomized and non-randomized trials have demonstrated activity in recurrent ovarian cancer for the alkylating agent treosulfan (Gropp et al. 1998; Köpf-Meier and Sass 1996; Meier et al. 2009). For patients in the palliative setting of recurrent ovarian cancer the maintenance of quality of life is, beside the maintenance of tumor control, the main goal of treatment. To sustain a maximum of flexibility, the choice of the route of administration is an important aspect in everyday life. Liu et al. (1997) assessed patient preferences for oral versus intravenous palliative chemotherapy using a structured interviewer-administered questionnaire. Major reasons for a clear preference for oral chemotherapy (92 of 103 patients) were convenience, problems with i.v. access and a better chemotherapy-taking environment outside the clinic. Nevertheless, despite their initial clear preference for oral chemotherapy patients were not willing to sacrifice efficacy for their preference. In contrast, the NOGGO study group has investigated the preference of elderly patients ≥65 years regarding p.o. or i.v. treosulfan for the treatment of relapsed ovarian cancer in 123 patients and showed a clear preference for the i.v. administration of treosulfan. The main reasons for this decision were the hope for less gastrointestinal toxicities, a better control of drug delivery and the assumption, that i.v. therapy is more effective. Therefore, in terms of different patient populations and their preferences the availability of different formulations might be worthwhile, if efficacy and safety are comparable. Here, we present the final results of our randomized study comparing p.o. and i.v. treosulfan to increase information on safety and efficacy of both formulations. Previous data especially on p.o. treosulfan were mainly based on a low number of patients. Detailed safety data were scarce. To our knowledge, our study is the largest performed in ovarian cancer comparing i.v. versus p.o. treosulfan in terms of efficacy and safety.

Despite the heavy pretreatment of the patients, treosulfan therapy was well tolerated in both treatment arms. The majority of toxicities were grade I or II. Most frequently observed hematological toxicities of grade III/IV in both treatment arms were leucopenia (9.2%; 3.9% in the i.v. arm and 14.8% in the p.o. arm) and thrombocytopenia (7.2%; 6.2% in the i.v. arm and 8.2% in the p.o. arm). Compared to the i.v. arm, a statistically significant higher number of grade III/IV leucopenia occurred in the p.o. arm in our study. This number might explain the higher number of treatment discontinuations in this treatment arm due to unacceptable toxicity. However, only one case of febrile neutropenia in the p.o. treatment arm was reported. Most frequent documented gastrointestinal toxicity grade III/IV was ileus (11.2%; 14.0% in the i.v. arm and 8.2% in the p.o. arm). Toxicities during therapy with intravenous treosulfan had been reported in several previous studies (Meier et al. 2009; Mahner et al. 2012). However, safety data on therapy with oral treosulfan are rare. Keldsen et al. (1998) reported that oral treosulfan treatment is well tolerated as second-line treatment for platinum-resistant cancer in a patient collective with a median age of 61 years. Meden et al. (1997) investigated toxicities of oral treosulfan in patients receiving treosulfan as maintenance therapy after first-line chemotherapy. As in our study, in total leucopenia and thrombocytopenia were among the most frequently reported grade III/IV toxicities. The frequency of observed thrombocytopenia (7.2%) is comparable with those published by Meier et al. (2009) (7.5%) and Mahner et al. (2012) (4.0%). In contrast, the frequency of leucopenia observed in the treosulfan i.v. arm in our study (3.9%) is much lower than that reported by Meier et al. (2009) (18.3%) and Mahner et al. (2012) (12.0%). Additionally neutropenia was far less frequently observed in our study (0.8%) than by Meier et al. (2009) (14.4%) and Mahner et al. (2012) (8.0%). Possible explanations for this difference in toxicity rates could be that Meier et al. used a more dose-dense treatment regimen (7000 mg/m^2^ d1 q3w), but patients in these study were less heavily pretreated.

No significant differences in disease control rate (41.4% in the i.v. arm and 36.9% in the p.o. arm), time to progression (3.7 months in the i.v. arm and 3.5 months in the p.o. arm) or overall survival (13.6 months in the i.v. arm and 10.4 months in the p.o. arm) were observed in our study. In comparison to data from a first-line study by Reed et al. (2006) and a second-line study by Gropp et al. (1998) on intravenous treosulfan, disease control (Reed: 50.0%; Gropp: 53.0%) and time to progression (Reed: 5.0 months) are slightly lower in our study. However, in our study we were dealing with patients with many concomitant diseases in more advanced treatment lines that were older than patients investigated in the second-line study by Gropp et al. In our study, a nonsignificant trend toward a prolonged survival in the i.v. arm could be observed (13.6 vs. 10.4 months). But this comparison is due the very limited methodological limitations only hypothesis generating.

Interestingly the number of previous treatment lines (two or less vs. more than two) did not have an impact on any of the efficacy parameters. This indicates that also heavily pretreated patients benefit from treosulfan treatment to the same extent. A recent large study of the German AGO stated that patients with relapsed ovarian cancer seem to benefit form subsequent relapse treatment at the second to fourth recurrence (Hanker et al. 2012).

Due to several amendments throughout the entire term of the study, different definitions of how to count previous treatment lines were used. As we merged induction and re-induction therapy for analyses, there might be an underestimation of previous therapy lines. This has to be considered when comparing our results with other studies.

When comparing survival data of platinum-resistant patients in our study to data from a large phase III study (Pujade-Lauriane et al. 2014) in which ovarian cancer patients are treated with chemotherapy ± bevacizumab in second and third line, overall survival of platinum-resistant patients treated with treosulfan in our study is within the same range as survival of platinum-resistant patients treated with chemotherapy only (11.9 vs. 13.3 months). Given the mild toxicity profile of treosulfan therapy and similar effect on disease survival, the treosulfan therapy seems to be an attractive option for treatment of the platinum-resistant recurrent disease.

The quality of life analysis did not show any significant differences in global health score or in selected subscales between the two treatment arms. In both arms a nonsignificant slight improvement in quality of life during therapy could be observed. However, due to many patients experiencing early progressive disease during therapy, the available data set shows a high dropout rate, limiting the meaningfulness of the analysis.

In summary, observed toxicities with oral and intravenous treosulfan were in the same range as in previous studies. Interestingly a higher number of leucopenia grade III/IV occurred in the p.o. treatment arm. Apart from this, no additional significant differences in toxicity were observed between the treatment arms. CBR, TTP and OS data were comparable to previously reported data and did not differ significantly between both treatment arms. Heavy pretreatment did not detract from the benefit of treosulfan treatment. Patients with ROC who have frequently more gastrointestinal symptoms and polypharmacy due to co-morbidities may tolerate an i.v. treatment better. In a synopsis of efficacy and safety outcomes, there might be an advantage for i.v. application form for treosulfan treatment. Still, treatment with oral treosulfan remains a considerable option.

## Electronic supplementary material

Below is the link to the electronic supplementary material.

Supplementary material 1 (DOCX 27 kb)

Supplementary material 2 (TIFF 33091 kb)
