# Supplementary material for: A phase III, open label, randomized multicenter controlled trial of oral versus intravenous treosulfan in heavily pretreated recurrent ovarian cancer: a study of the North-Eastern German Society of Gynecological Oncology (NOGGO)
Source: J Cancer Res Clin Oncol. 2016 Nov 28;143(3):541–50. doi: 10.1007/s00432-016-2307-0 (PMC5306340; doi:10.1007/s00432-016-2307-0)
Supplement: Supplementary file 1 — Supplementary material 1 (DOCX 27 kb) [file 432_2016_2307_MOESM1_ESM.docx]

S1:

**A** Clinical benefit rate (CBR)

|  |  |  | Logistic model | | |
| --- | --- | --- | --- | --- | --- |
| Factor | Category | N | CBR (%) | OR (95% CI) | p |
| Route of administration | Oral | 122 | 36.9 |  | 0.522 |
|  | i.v. | 128 | 41.4 |  |  |
|  |  |  |  |  |  |
| Platinum sensitivity | Sensitive | 117 | 47.9 | 2.30  (1.34 – 3.99) | 0.003 |
|  | Resistant | 133 | 31.6 |  |  |
|  |  |  |  |  |  |
| Number of previous therapy lines | >2 | 89 | 41.6 |  | 0.368 |
|  | ≤2 | 161 | 37.9 |  |  |
|  |  |  |  |  |  |
| Karnofski Index | <90 | 92 | 45.1 |  | 0.070 |
|  | ≥90 | 133 | 33.7 |  |  |
|  | missing | 25 |  |  | 0.061 |

**B** Time to progression (TTP)

|  |  |  | Cox model | | |
| --- | --- | --- | --- | --- | --- |
| Factor | Category | N | TTP | HR (95%CI) | p |
| Route of administration | Oral | 122 | 3.5 |  | 0.506 |
|  | i.v. | 128 | 3.7 |  |  |
|  |  |  |  |  |  |
| Platinum sensitivity | Sensitive | 117 | 5.0 | 0.46  (0.34 – 0.63) | <0.0001 |
|  | Resistant | 133 | 2.8 |  |  |
|  |  |  |  |  |  |
| Number of previous therapy lines | >2 | 89 | 3.4 |  | 0.587 |
|  | ≤2 | 161 | 3.3 |  |  |
|  |  |  |  |  |  |
| Karnofski Index | <90 | 92 | 3.0 | 1.38  (1.02 – 1.86) | 0.039 |
|  | ≥90 | 133 | 4.3 |  |  |
|  | missing | 25 |  | 1.68  (1.00 – 2.80) | 0.048 |

**C** Overall Survival (OS)

|  |  |  | Cox model | | |
| --- | --- | --- | --- | --- | --- |
| Factor | Category | N | OS | HR (95%CI) | p |
| Route of administration | Oral | 122 | 10.4 |  | 0.065 |
|  | i.v. | 128 | 13.6 |  |  |
|  |  |  |  |  |  |
| Platinum sensitivity | Sensitive | 117 | 12.8 |  | 0.156 |
|  | Resistant | 133 | 11.9 |  |  |
|  |  |  |  |  |  |
| Number of previous therapy lines | >2 | 89 | 10.6 |  | 0.228 |
|  | ≤2 | 161 | 12.9 |  |  |
|  |  |  |  |  |  |
| Karnofski Index | <90 | 92 | 8.6 | 1.93  (1.41 – 2.64) | <0.0001 |
|  | ≥90 | 133 | 15.1 |  |  |
|  | missing | 25 |  |  | 0.190 |
