# Supplementary figures and images for: A phase III, open label, randomized multicenter controlled trial of oral versus intravenous treosulfan in heavily pretreated recurrent ovarian cancer: a study of the North-Eastern German Society of Gynecological Oncology (NOGGO)
Source: J Cancer Res Clin Oncol. 2016 Nov 28;143(3):541–50. doi: 10.1007/s00432-016-2307-0 (PMC5306340; doi:10.1007/s00432-016-2307-0)

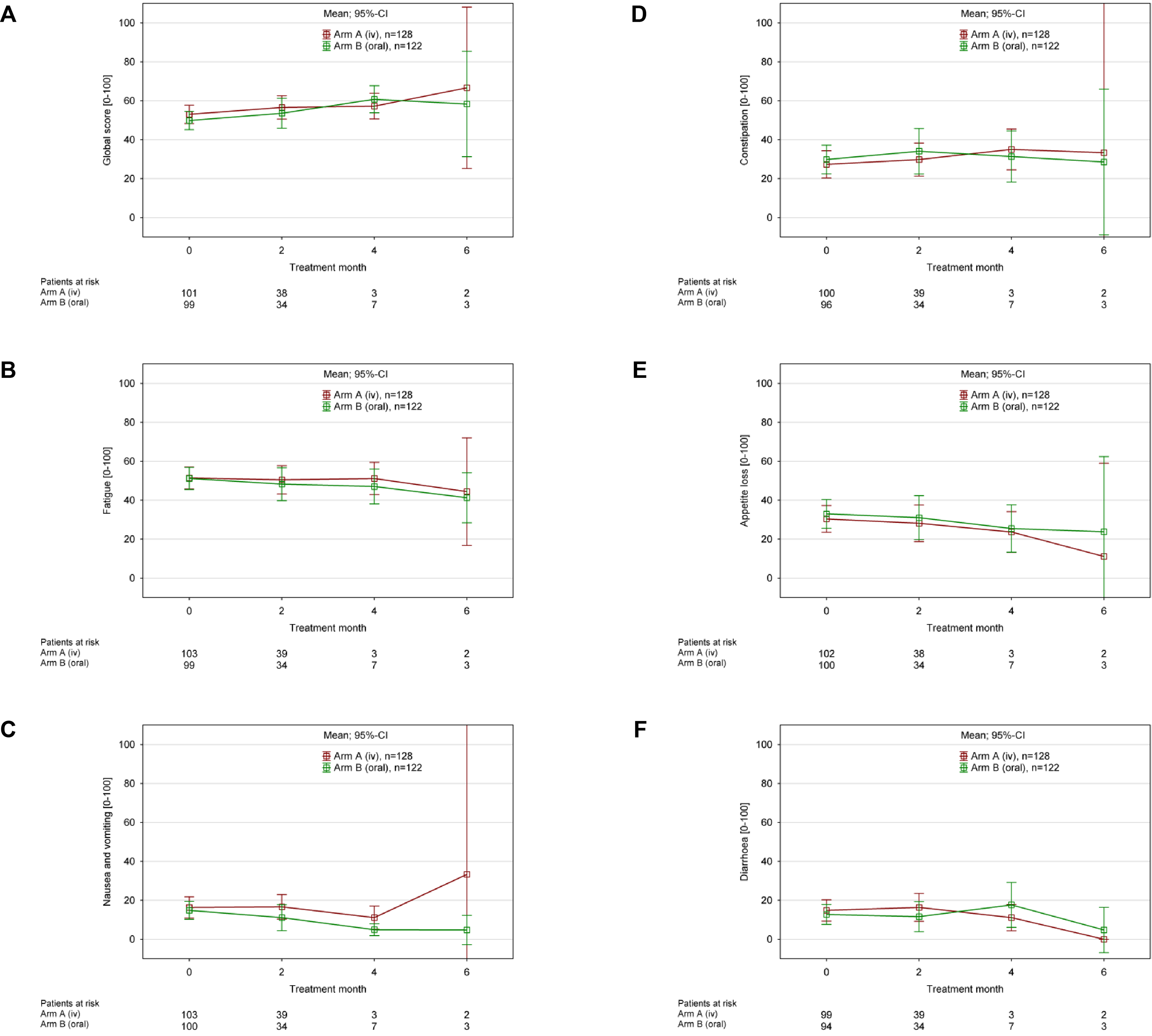

Supplement: Supplementary file 2 — Supplementary material 2 (TIFF 33091 kb) [file 432_2016_2307_MOESM2_ESM.tif]
